# Principles of Valgus Intertrochanteric Osteotomy (VITO) after Femoral Neck Nonunion

**DOI:** 10.1155/2018/5214273

**Published:** 2018-12-02

**Authors:** D. Banaszek, D. Spence, P. O'Brien, K. Lefaivre

**Affiliations:** Vancouver General Hospital, UBC Department of Orthopaedics, Division of Orthopaedic Trauma, Vancouver, BC, Canada

## Abstract

Nonunion is a relatively rare, yet challenging problem after fracture of the femoral neck. Risk factors include verticality of the fracture line and presence of comminution of the posteromedial calcar, as well as quality of reduction. Treatment options consist of valgus intertrochanteric osteotomy versus arthroplasty. Treatment should be tailored to the individual patient, taking into account patient age and activity demands. This review outlines the principles and technical considerations for valgus osteotomy of the proximal femur in the setting of femoral neck nonunion.

## 1. Risk Factors for Femoral Neck Nonunion

### 1.1. Definition of Nonunion

An understanding of the pathogenesis for any condition requires a precise definition of diagnostic criteria. The US Food and Drug Administration (FDA) thereby defines a “fracture nonunion” as a fracture which is at least 9 months old and does not show radiographic evidence of healing for 3 consecutive months [1]. Previous literature has established, however, that healing metrics may be different depending on multiple patient and fracture characteristics, including fracture location [2]. Perhaps a more clinically applicable definition, as set forth by Calori et al., is a fracture with little or no possibility of healing without further intervention [1].

Diagnosis of a nonunion is most commonly established on standard radiography, but often confirmed using advanced imaging modalities. Specific work-up as to the primary cause of nonunion should be individualized to the particular patient based on history and physical examination. An infectious etiology should be ruled out with physical and laboratory signs, including history of fevers/chills, erythema, or fluctuance to previous surgical wounds, as well as a complete blood cell count with differential, ESR/CRP, and site-specific or blood cultures as appropriate. Metabolic disease necessitates endocrinologic, as well as nutritional work-up. Correlations between low calcium and vitamin D levels have been observed in a multitude of animal studies [3, 4]. Reports have been more controversial in humans, however [5–8]. A recent systematic review concluded that although vitamin D has a role in fracture healing, data remain too inconsistent to elucidate any conclusions for treatment [9].

### 1.2. Patient and Injury Characteristics

Specific nonunion after femoral neck fracture is a unique and challenging problem facing the treating orthopaedic surgeon. Literature is relatively scarce on the overall incidence and risk factors for nonunion in younger patients, as a large proportion of published series denote rates in the elderly. Femoral neck fractures tend to occur in a bimodal age distribution, the majority of which are in an elderly population as a result of low energy falls. This is in comparison to younger patients, who suffer femoral neck fractures most often due to high-energy trauma [10, 11].

Differences in mechanism between young and elderly patients suffering fractures of the femoral neck present unique challenges in treatment. The young patient is more likely to present with associated injuries to other parts of the musculoskeletal system, as well as different organ systems. Unfortunately, the link between mechanism and risk of nonunion has been difficult to establish, due to heterogeneity in definitions. Nonetheless, age has been identified as an independent risk factor for femoral neck nonunion in multiple series. One study examining nonunion rate after femoral neck internal fixation noted an overall incidence rate of nonunion of 19.3%, although rates in patients aged 40 were far lower (5.9%) as compared to patients over the age of 70 (24.9%). Incidence rates increased with each decade of life [10]. A more recent study looking at a prospective series of 106 patients under the age of 60 noted a similar rate of 5.6% (6 patients) with femoral neck nonunion after attempted internal fixation [12].

### 1.3. Fracture Characteristics

Fracture characteristics are thought to play a major role in the risk of nonunion as well. More specifically, rate of union has been studied with regard to fracture location, morphology, and characteristics of treatment. In a historical series, Dedrick et al. reported an overall nonunion rate of 20%. Subcapital fractures, however, witnessed an 83% rate of nonunion or avascular necrosis, versus only 21% with true transcervical femoral neck fractures. There was no difference in nonunion rate in terms of cause of injury, degree of comminution, treatment method, or prior health status [13]. Similarly, multiple series have reported on fracture displacement as a significant predictor of nonunion. Parker et al. found a significantly increased rate of nonunion in displaced (30.1%) versus nondisplaced (8.5%) femoral neck fractures in a series of 1133 patients [10]. A different series examining risk of nonunion by Garden classification noted rates of 6, 18, 23, and 38% for Garden grades I, II, III, and IV, respectively [14]. A meta-analysis of 564 fractures noted an overall nonunion rate of 8.9%, but with increased rates in displaced fractures treated with open versus closed reduction [15].

**Figure 1:**
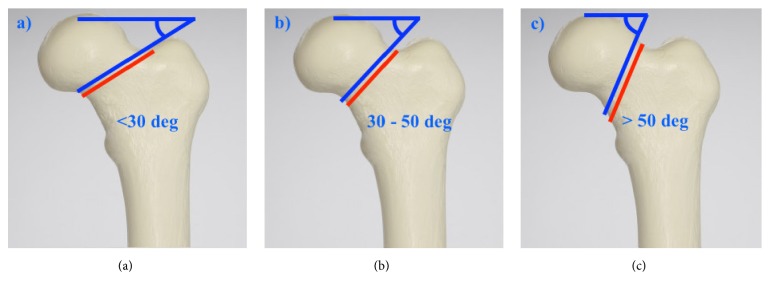
Pauwels classification of fracture line obliquity. (a) Grade I, <30 degrees, (b) Grade II, 30 – 50 degrees, and (c) grade III, >50 degrees.

Pauwels et al. initially described a classification system based on the verticality of the fracture line of the femoral neck [16, 17] (Figure 1). The increase in obliquity of the fracture line equates to an increase in shear forces across the fracture site with a concomitant decrease in compression forces. Previous series have identified high Pauwels grade as a risk factor for femoral neck nonunion [18–20].

### 1.4. Operative Intervention and Timing

To date, no clear consensus has been established in terms of operative strategies to address young patients with femoral neck fractures in terms of timing, implant choice, or open versus closed treatment [21]. Nonetheless, variables as related to surgical treatment of femoral neck fractures have been studied as predictors of nonunion [22]. Previous series have noted increased rates of complications in fractures with fixation delayed over 12 hours [23]. To date, the only level-1 study directly addressing the question of fracture fixation timing showed no differences in nonunion rates between fractures fixed in less than versus more than 48 hours [24]. Major contributors to nonunion risk in this study included posterior comminution, poor radiographic reduction, and improper screw placement. Damany et al. similarly showed no difference in nonunion rate with fractures treated in less than 12 versus greater than 12 hours [15]. In a series of 202 patients, Yang et al. noted a decreased nonunion rate with inverted triangle-style cannulated screw configuration for femoral neck fracture fixation. Fracture displacement and quality of reduction parameters were also predictive of nonunion, and need for future revision arthroplasty procedure [11]. A recent comparative trial examining the use of cannulated screw fixation versus sliding hip screw found a slight advantage to use of sliding hip screws in displaced fractures, but no overall difference between implants otherwise [25, 26].

## 2. Treatment Principles

Surgical treatment after clinical and radiographic confirmation of femoral neck nonunion should be individualized to the particular patient. Age, activity level, bone quality, and metabolic disturbances, as well as patient modifiable factors such as weight bearing compliance and smoking must be taken into account when choosing the appropriate procedure [14, 21]. The presence of advanced avascular necrosis with femoral head collapse, poor bone stock, and advanced age should prompt the surgeon to discuss arthroplasty as the optimal treatment [35]. Valgus intertrochanteric osteotomy is reserved for highly active patients with quality remaining bone stock who are able to comply with postoperative restrictions.

### 2.1. Compromise of Future THA?

To our knowledge, no studies have specifically looked at revision arthroplasty for failed VITO. Undoubtedly, the altered shape of the proximal femur makes this procedure more difficult, however. Although no specific series has commented on conversion to arthroplasty after VITO for femoral neck nonunion, several authors have examined THA after failed osteotomy for primary hip osteoarthritis. Results are generally reported as inferior as compared to total hip arthroplasty for primary osteoarthritis. In a retrospective series of 305 arthroplasties, early and late complications were reported as 11.8% and 13.1%, respectively. 18.1% of hips had been revised at 10-year follow-up [36]. Another series noted an average of 14 years between VITO and THA conversion in 30 hips, with improved results with cemented versus uncemented components [37]. Despite challenges and a higher complication rate, arthroplasty conversion can nonetheless be performed with good results.

### 2.2. VITO Principles

The original description of valgus reorientation osteotomy of the proximal femur was described by Pauwels et al. in the 1930s [16]. The basic biomechanical tenet of this technique is the redirection of forces to create a more favourable fracture healing environment. More specifically, due to the anatomy of the proximal femur, high-grade Pauwels (i.e., vertically oriented) fractures prone to a shearing force are converted to more horizontal compressive forces when subjected to patient load.

**Figure 2:**
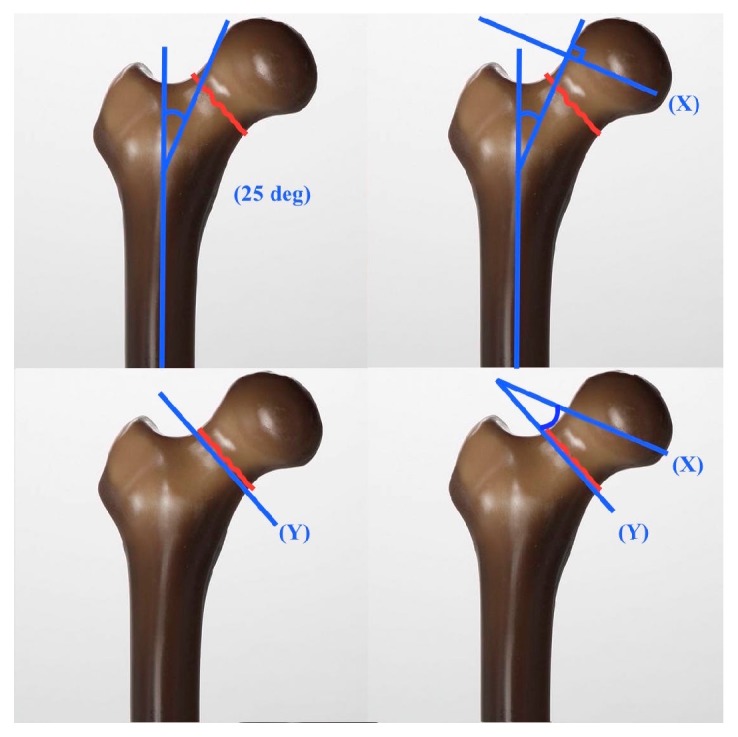
Preoperative planning for valgus intertrochanteric osteotomy (VITO). A line representing the compressive force is drawn 25 degrees from the anatomical axis of the femur (upper left). A line perpendicular to the compressive force (X) is then drawn for reference (upper right). Line (Y) represents fracture line obliquity (lower left). The angle formed between (X) and (Y) will reflect the angle of the resected wedge (lower right).

Preoperative planning is carefully conducted to determine both the size of the resected wedge, as well as implant positioning. The initial description by Pauwels claimed a 25-degree orientation of the nonunion site relative to the patient's femoral anatomic axis to achieve an optimal healing environment. The appropriate closing wedge resection is then planned based on an angle formed between a reference and the verticality of the fracture, at or slightly above the level of the lesser trochanter (Figure 2). Classically, templating is performed using a goniometer and tracing paper, although modern templating software is also available (TraumaCad ®; Brainlab, Munich Germany).

Multiple implant types for fixation have been described, including angled blade plates (ABPs), the dynamic hip screw (DHS), and the dynamic condylar screw (DCS). The classic description using the ABP offers the theoretical advantage of less bony resection of the femoral neck, and less iatrogenic avascular necrosis [12]. In this approach, preoperative templating is used using a 95-degree angled blade plate, using a chisel through the lateral proximal femoral cortex directed into the femoral head to carve a path for the blade plate. With the popularization of this technique, manufacturers have expanded plate options to include a multitude of angles, thus allowing for more freedom of correction. This technique is nonetheless technically challenging, however, as it does not allow for any rotational margin of error of the implant.

Advantages of the DCS plate include the relative technical ease of the procedure, as well as the ability to compress the nonunion site through the large proximal lag screw. Furthermore, at the author's institution, this plate is bent according to preoperative plans, allowing for improved contact throughout the lateral cortex after osteotomy completion [38].

## 3. Surgical Technique and Case Presentation

**Figure 3:**
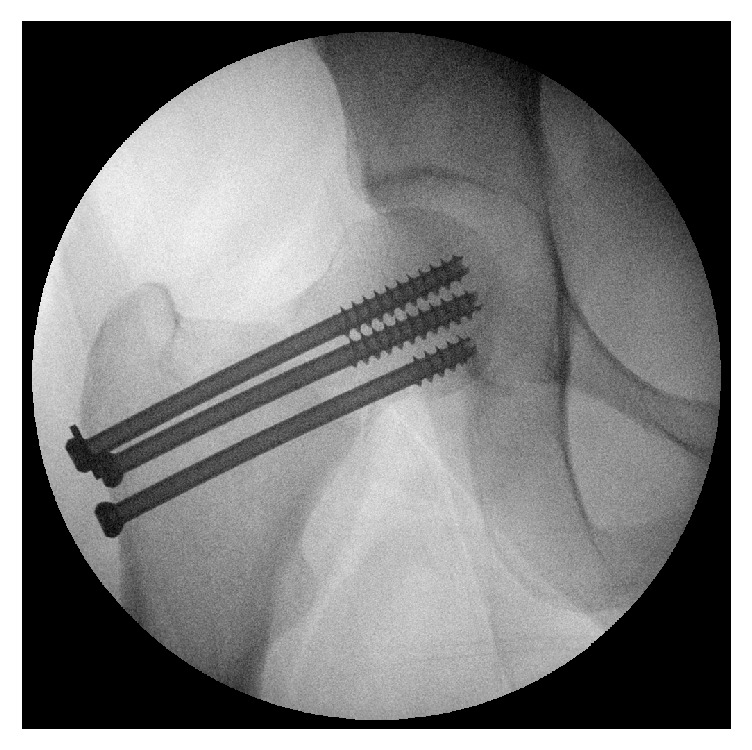
Intraoperative views of initial femoral neck fracture fixation using an inverted triangle configuration of 3 cannulated screws.

**Figure 4:**
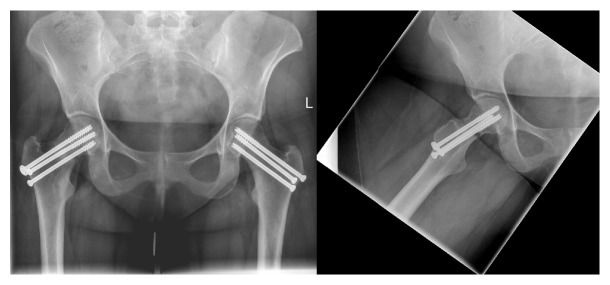
Radiographic evidence of femoral neck fracture nonunion, taken at 10 months postoperatively.

**Figure 5:**
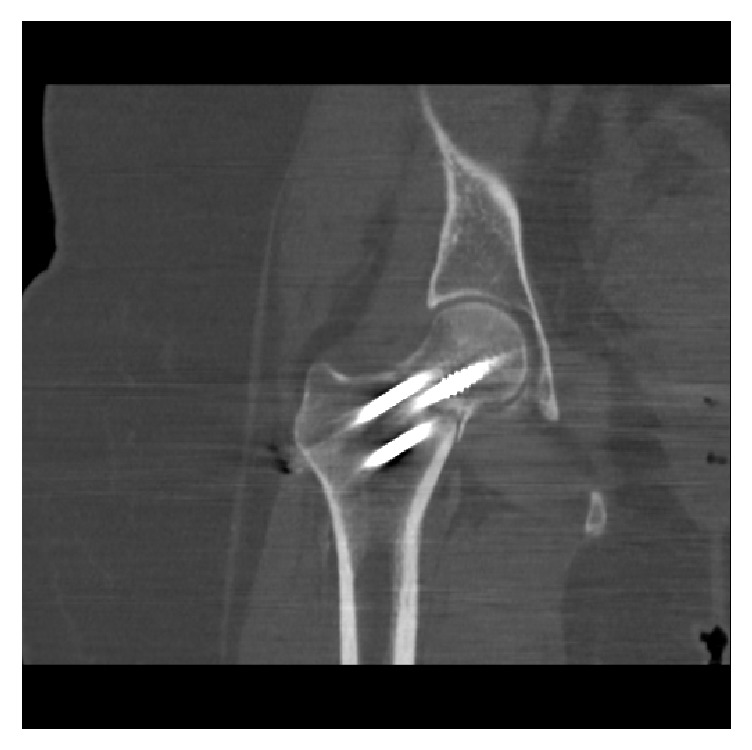
Computer tomography confirmation of nonunited femoral neck fracture.

**Figure 6:**
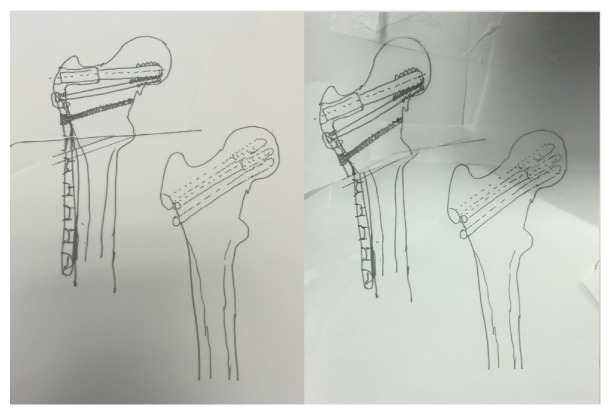
Preoperative templating for patient including 20-degree wedge. Images represent planning before (left) and after (right) simulated wedge resection from tracing paper.

The presented case is that of an otherwise healthy 24-year-old female who suffered bilateral stress fractures of the femoral necks. Initial fixation was conducted bilaterally with cannulated screws in an inverted triangle configuration (Figure 3). Despite satisfactory results on the contralateral left side, the patient continued to complain of hip and groin pain on the right, with subsequent radiographs (Figure 4) and computer tomography scanning (Figure 5) confirming the presence of a nonunion. Clinical exam revealed an antalgic gait with limitations in flexion and internal rotation of the hip. Initial work-up excluded the presence of infection, nutritional problem, or calcium and vitamin D deficiency. Given the patient's increased BMI, clinical suspicion of repeated stress in the femoral neck region was the accepted explanation for initial treatment failure. Preoperative planning called for a 20-degree wedge resection of the lateral proximal femur to optimize the load across the fracture. The authors would recommend a maximum if 40 degrees of correction is given the associated soft tissue tightness and propensity for leg abduction at higher levels of correction (Figure 6).

**Figure 7:**
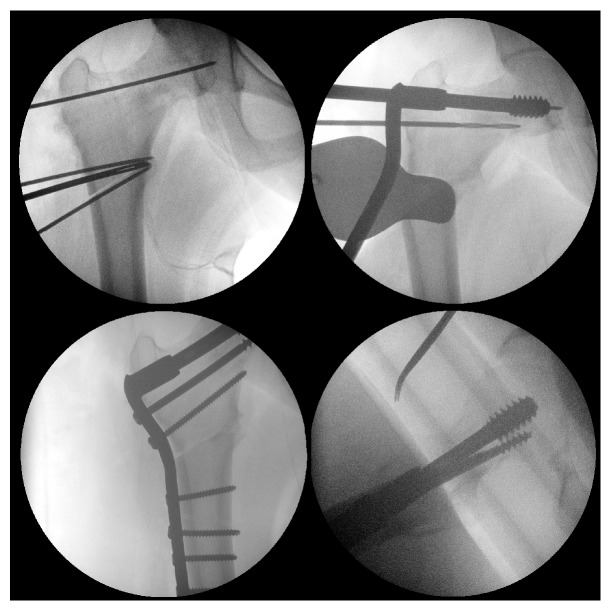
Intraoperative views of valgus intertrochanteric osteotomy (VITO) osteotomy technique at author's center. Panels showing initial DCS guidewire placement and planning of osteotomy wedge (upper left), positioning of DCS lag screw with prebent plate (upper right), and anteroposterior (lower left) and lateral (lower right) images of compressed osteotomy site.

The patient was positioned supine on a fracture table with the operative limb in traction. After initial removal of the implants, an extensile lateral approach to the lateral femur was utilized. The initial DCS lag screw guide-pin, using the standard guide on the lateral femur, was then inserted across the nonunion site, overdrilled, and the lag screw inserted under manual power. Guidewires were placed proximally and distally to the planned osteotomy site both for saw guidance as well as rotational alignment. The standard ABP set has stainless steel triangles at multiple angles that are optimal for this purpose. The 9-hole DCS plate was bent intraoperatively over the large bending press to 20 degrees at the templated level. This technique of prebending the plate is used at our institution due to ease of application over the lateral femoral cortex, but placement of the lag screw off axis without bending with subsequent rotation of the plate to the lateral cortex is also possible. The osteotomy was then carefully completed under fluoroscopic guidance, first using multiple k-wire holes to avoid saw necrosis along the osteotomy path, and completed with an osteotome. The DCS plate was then secured proximally over the lag screw, with an additional lag screw placed slightly inferiorly also across the nonunion site. Control of the distal shaft was obtained via large bone forceps. The osteotomy site was then compressed using the articulated tensioning device from the large fragment bone set and secured using large fragment cortical screws into the distal femoral shaft. Final fluoroscopic views were taken to assure accurate implant position and osteotomy compression (Figure 7).

**Figure 8:**
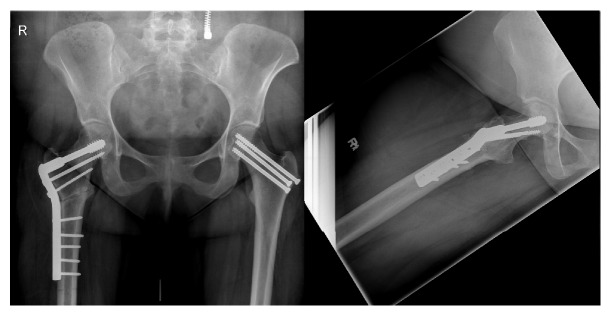
4-week follow-up after VITO procedure.

**Figure 9:**
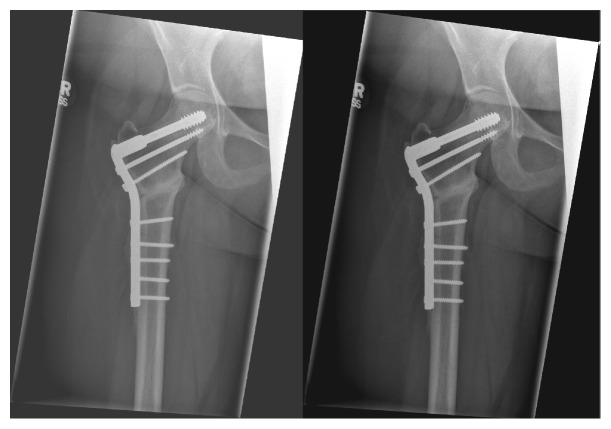
6-month (left) and 1-year (right) follow-up after VITO procedure.

Postoperatively, the patient was made touch-down weight bearing for a period of six weeks, with four weeks of DVT prophylaxis. Regular follow-up revealed healed nonunion and osteotomy sites and a good clinical result at 4 weeks (Figure 8), 6 months, and 1 year (Figure 9).

## 4. Discussion

### 4.1. Results of VITO

The evidence on valgus intertrochanteric osteotomy for femoral neck nonunion is limited to retrospective series. Despite some variation in operative technique, the majority of authors report good-to-excellent outcomes with this procedure. A summary is provided in Table 1.

Marti et al. reviewed 44 hips after Pauwels abduction osteotomy with average follow-up of 7.1 years. Seven patients required conversion to THA in total. Despite 22 hips showing evidence of femoral head osteonecrosis, only three of these patients were among those converted to total hip arthroplasty. In patients who did require arthroplasty, the average Harris Hip Score was 91 [28].

Min et al. achieved union in 9/11 cases with an average of 12.5 weeks. Total follow-up was 4.9 years. Functional outcomes were reported as excellent in nine patients, with the other two patients undergoing total hip arthroplasty for femoral head avascular necrosis [27].

Magu et al. reported successful union in 44 of 48 hips after VITO procedure. Of the remaining 4 hips, 2 went on to successful union after revision osteotomy. This group witnessed only two reported cases of avascular necrosis with average follow-up of 6 years. Statistical improvements were made in terms of neck shaft angle, and limb-length discrepancy. Harris Hip scores averaged to 86.7, and 40 of 48 patients had near-normal gait pattern [30].

Gupta et al. reported successful radiographic union of 56/60 patients treated with Pauwels-style osteotomy after neglected femoral neck fracture. Fracture line Pauwels angle was corrected from an average of 65 degrees to 26 degrees. Average time to union was 3.9 months, with a follow-up of 3.5 years [31].

Said et al. reviewed 36 patients with either nonunion or neglected femoral neck fracture, with union achieved in 35 (97%) after VITO osteotomy. Average time to union was 4 months. 61% of patients achieved pain-free gait, with 25% having pain with prolonged activity and 14% having persistent pain. Average limb-length discrepancy was changed from 2.5 cm preoperatively to 0.5 cm [32].

Magu et al. reviewed 39 patients, achieving union in 9/11 with primary VITO procedure and 27/28 with failed dynamic hip screw or lag screw fixation. Limb length was corrected in 14/16 patients with preoperative discrepancy. Modified Harris Hip scores averaged 85.6, with good-to-excellent functional outcomes achieved in 32/39 patients [33].

Varghese et al. reported on 32 consecutive patients, with union achieved for 29 (91%) in 6+/-7 months. Harris Hip scores averaged 82 ± 13 points. Poor functional outcome was associated with increased valgus alignment of greater than 15 degrees as compared to the contralateral side. The presence of avascular necrosis had no statistical association with outcome [34].

Kumar et al. examined 50 cases of neglected femoral neck fractures, with bony union achieved in 45 (90%) after modified Pauwels osteotomy. Excellent results using Askin and Bryan's criteria were seen in 35 patients, and poor results seen in 5 patients. Of the patients with poor results, three had a persistent nonunion, one implant breakage, and one implant cut-out. Two of the patients underwent revision osteotomy, one underwent total hip arthroplasty, and two refused revision surgery [29].

Finally, Yuan et al. aimed to establish whether slight undercorrection of the planned resection wedge could achieve results similar to a full planned resection. Twenty-five patients underwent 20-degree wedge resection and were compared to seven patients who underwent 30 degree resections. There were no differences in terms of union between the two cohorts, with only a single patient going on to persistent nonunion. Patients who underwent a thirty-degree resection were more likely to develop avascular necrosis, however (67% versus 12%) [37].

## 5. Conclusion

Valgus intertrochanteric osteotomy is the treatment of choice for active patients aged less than 50 years with a nonunion of a femoral neck fracture. This technique requires precise preoperative planning and intraoperative skill. Implant selection includes angled blade plates (ABPs), dynamic hip screws (DHS), and dynamic condylar screw (DCS) plates. Union rates approach 90% in most reported series. Patients should be warned of the risk of avascular necrosis, as well as the difficulty in performing revision arthroplasty in this setting.

## Figures and Tables

**Table 1:** Results of VITO.

Authors	Study Type	Treatment	Union Rate	Complications
Min et al. [27]	Retrospective Review 11 pts.	ABP and Valgus Osteotomy (VO)	9/11	2/11 AVN, requiring THA

Marti et al. [28]	Retrospective Review 50 pts.	ABP and VO	43/50	7/50 AVN requiring THA 6/50 required re-operation

Kumar et al. [29]	Retrospective Review 50 pts.	ABP and VO	45/50	1/50 nonunion requiring THA 2/50 revision osteotomy 2/50 refused revision

Magu et al. [30]	Retrospective Review 48 pts.	ABP and VO	44/48	2/48 revision osteotomy 2/48 AVN

Gupta et al. [31]	Retrospective Review 60 pts.	ABP and VO	56/60	2/60 revision osteotomy 2/60 AVN

Said et al. [32]	Retrospective Review 36 pts.	ABP and VO	35/36	1/36 requiring THA Nonunion

Magu et al. [33]	Retrospective Review 39 pts.	ABP/SHS and VO	44/48	4/48 nonunion

Varghese et al. [34]	Retrospective Review 32 pts.	ABP and VO	29/32	3/32 nonunion

## Data Availability

No external data is applicable to this manuscript.
